# Supplementary material for: Determinants of early initiation of breastfeeding following birth in West Africa: A multilevel analysis using data from multi-country national health surveys
Source: PLoS One. 2024 May 16;19(5):e0302143. doi: 10.1371/journal.pone.0302143 (PMC11098389; doi:10.1371/journal.pone.0302143)
Supplement: Attachment — Submitted filename: Comments.docx [file pone.0302143.s002.docx]

1. The objectives and rationale of the study were clearly stated. However, the abstract word count is higher than expected. I believe it is too much. The abstract should be reduced to an acceptable word count.
2. Authors should accurately state the purpose of the intraclass correlation coefficient (ICC), median odds ratio (MOR), and proportional change in variance (PCV) as they are random components and are not used to evaluate model fitness.
3. In the abstract section, the authors mention that they have used the kids recorded data set, but they also report that they have used combined data from the women's files. This needs clarification.
4. The authors should involve the sample size determination procedure via table, graph, or diagram that was used and should be referenced as well as clearly stated.
5. Authors should state both the dependent and independent variables in tabular form for easy comprehension by readers who will eventually have access to them in the long run.
6. Weighting DHS data for a single country is different from weighting DHS data for multicounty analysis. Even though the authors used DHS data from 13 countries, they used sampling weight, which is used for a single-country DHS data weighting technique. Instead, the authors have to use other weighting techniques, for instance, differential weighing...
7. DHS data may have different formats, standards, definitions, or classifications that make them difficult to compare, link, or aggregate across countries. How do authors handle the above challenges during multicounty analysis?
8. It is good that the authors appended the West African countries DHS data for secondary analysis, but the big concern is how authors address the effect of the variation of time to combine data sets for analysis.
9. Cluster analysis have to conducted to identify patterns and trends in the data, such as the characteristics and behaviors of different segments of the population; explore the heterogeneity and diversity of the data; evaluate the impact and effectiveness of interventions or programs, such as the differences in outcomes or indicators across clusters; and inform policy and decision-making, such as the allocation of resources or the targeting of interventions to specific clusters.
10. Did the authors do sensitivity analysis? If not, I recommend the authors do a sensitivity analysis. Because it is essential to testing the robustness and reliability of the results of a data analysis by varying the assumptions, parameters, or data used in the analysis. Additionally Sensitivity analysis can help the authors to assess the impact of uncertainty, variability, or bias on the estimates or conclusions of their analysis.
11. The results of the outcome variable and some independent variables are better represented in figures or charts than in tables.
