# Supplementary material for: Determinants of early initiation of breastfeeding following birth in West Africa: A multilevel analysis using data from multi-country national health surveys
Source: PLoS One. 2024 May 16;19(5):e0302143. doi: 10.1371/journal.pone.0302143 (PMC11098389; doi:10.1371/journal.pone.0302143)
Supplement: Attachment — Submitted filename: Response to Reviewers Comments.docx [file pone.0302143.s004.docx]

Response to the Review Comments

**Dear editor and reviewers**

**First for all the authors would like to thank the editor(s) and reviewers for your consideration, precious time, thoughtful comments and constructive suggestions, which help to improve the quality of this manuscript. We have responded to each critique/ comment and believe that the manuscript is much improved with the changes we made as suggested by the editor and reviewers. The corresponding changes and refinements made in the revised manuscript are summarized in our response below.**

1. Is the manuscript technically sound, and do the data support the conclusions?

The manuscript must describe a technically sound piece of scientific research with data that supports the conclusions. Experiments must have been conducted rigorously, with appropriate controls, replication, and sample sizes. The conclusions must be drawn appropriately based on the data presented.

Reviewer #1: Yes

Reviewer #2: Yes

**Response: Dear reviewers, thank you very much for your feedback and cross validation for our work. Please see our detail response below. Thank you** 2. Has the statistical analysis been performed appropriately and rigorously?

Reviewer #1: Yes

Reviewer #2: Yes

**Response: Dear reviewers, thank you very much for your feedback and cross validation for our work. Please see our detail response below. Thank you** 3. Have the authors made all data underlying the findings in their manuscript fully available?
The [PLOS Data policy](http://www.plosone.org/static/policies.action#sharing) requires authors to make all data underlying the findings described in their manuscript fully available without restriction, with rare exception (please refer to the Data Availability Statement in the manuscript PDF file). The data should be provided as part of the manuscript or its supporting information, or deposited to a public repository. For example, in addition to summary statistics, the data points behind means, medians and variance measures should be available. If there are restrictions on publicly sharing data—e.g. participant privacy or use of data from a third party—those must be specified.

Reviewer #1: Yes

Reviewer #2: Yes

**Response: Dear reviewers, thank you very much for your feedback and cross validation for our work. Please see our detail response below. Thank you** 4. Is the manuscript presented in an intelligible fashion and written in standard English?
PLOS ONE does not copyedit accepted manuscripts, so the language in submitted articles must be clear, correct, and unambiguous. Any typographical or grammatical errors should be corrected at revision, so please note any specific errors here.

Reviewer #1: Yes

Reviewer #2: Yes

**Response: Dear reviewers, thank you very much for your feedback and cross validation for our work. Please see our detail response below. Thank you**

**Reviewer #1:**

 This study was done before the COVID-19 pandemic. It would be very beneficial if there were also data referring to post-pandemic era. Kindly see comments in the uploaded word document. thank you so much.

**Response: Dear Reviewer, thank you for your feedback on our study. We appreciate your suggestion to include data referring to the post-pandemic era. We understand the value of examining the potential impact of the COVID-19 pandemic on our study's subject matter. However, we would like to clarify that our study is based on the data from the DHS (Demographic and Health Surveys), as a result, we do not have access to data specifically related to the post-pandemic era within the scope of our study. While we acknowledge the importance of investigating the potential effects of the pandemic on the topic of our study, it would require a separate research effort and data collection specifically focused on the post-pandemic period. We appreciate your thorough review and the comments provided in the uploaded word document, and we will address them accordingly to improve the quality and clarity of our manuscript. Thank you once again for your valuable feedback.**

1. The objectives and rationale of the study were clearly stated. However, the abstract word count is higher than expected. I believe it is too much. The abstract should be reduced to an acceptable word count.

**Response: Dear Reviewer, thank you for your valuable feedback regarding our study. We appreciate your acknowledgment that the objectives and rationale of the study were clearly stated. We understand your concern regarding the word count of the abstract exceeding expectations. We carefully reviewed the abstract and make necessary revisions to reduce its word count while ensuring that all essential information is retained. Our aim is to present a comprehensive overview of the study's key findings, methodology, and implications within the acceptable word count limits. We focused on condensing the abstract without compromising the clarity and coherence of the content. By doing so, we believe we can effectively communicate the core aspects of the study to readers while adhering to the required word count.**

2. Authors should accurately state the purpose of the intraclass correlation coefficient (ICC), median odds ratio (MOR), and proportional change in variance (PCV) as they are random components and are not used to evaluate model fitness.

**Response: Dear reviewer, thank you for your feedback on our study. We appreciate your comments regarding the accurate statement of the purpose of certain statistical measures, namely the intraclass correlation coefficient (ICC), median odds ratio (MOR), and proportional change in variance (PCV). We apologize for any confusion or misinterpretation in our manuscript. We understand that these measures are random components and are not typically used to evaluate model fitness. Instead, they serve different purposes in statistical analysis. To rectify this issue, we revised our manuscript to provide a clear and accurate explanation of the intended purposes of these measures. We ensured that the correct interpretation and use of these statistical measures are presented, aligning with the established literature and best practices.**

3. In the abstract section, the authors mention that they have used the kids recorded data set, but they also report that they have used combined data from the women's files. This needs clarification.

**Response: Dear Reviewer, thank you for your feedback on our study. We appreciate your observation regarding the mention of using both the kids recorded (KR) dataset and combined data from the women's files in the abstract section. To clarify, we would like to confirm that we have only utilized the kids recorded (KR) dataset for our analysis. In our revised manuscript, we made sure to accurately state that our study exclusively relies on the kids recorded (KR) dataset. We also provided a more precise and clear description of the dataset used in our methods (data source subsection) to avoid any confusion or ambiguity.**

4. The authors should involve the sample size determination procedure via table, graph, or diagram that was used and should be referenced as well as clearly stated.

**Response: Dear reviewer, thank you for your feedback on our study. We appreciate your suggestion to involve the sample size determination procedure in a more explicit manner, using a table, graph, or diagram, and referencing it appropriately. We agreed that providing clear information about the sample size determination procedure is essential for transparency and reproducibility. In our revised manuscript, we incorporated a dedicated section or subsection that explicitly outlines the sample size determination procedure. This visual representation will enable readers to better understand the considerations and methods employed to arrive at the chosen sample size. Thank you for your valuable input.**

5. Authors should state both the dependent and independent variables in tabular form for easy comprehension by readers who will eventually have access to them in the long run.

**Response: Dear reviewer, thank you for your feedback on our study. We appreciate your suggestion to present the dependent and independent variables in tabular form to facilitate easy comprehension for readers, both in the immediate context and in the long run. We agreed that tabulating the variables would provide a clear and concise overview of the variables included in our analysis. In our revised manuscript, we incorporated a table that presents the dependent and independent variables used in the study. Thank you for your valuable suggestion.**

6. Weighting DHS data for a single country is different from weighting DHS data for multicounty analysis. Even though the authors used DHS data from 13 countries, they used sampling weight, which is used for a single-country DHS data weighting technique. Instead, the authors have to use other weighting techniques, for instance, differential weighing...

**Response: Dear reviewer, thank you for your feedback on our study. We appreciate your comment regarding the weighting technique used in our analysis of the DHS data from multiple countries. We understand your suggestion to consider alternative weighting techniques, such as differential weighing, for multi-country analyses. However, it is important to note that the use of sampling weights provided by the DHS program is a widely accepted and recommended practice for analyzing DHS data across multiple countries. The DHS program provides explicit guidelines and recommendations on the appropriate use of sampling weights for multi-country analyses. These weights are designed to account for the complex survey design and ensure that the results are representative at the national and subnational levels.**

**While other weighting techniques, such as differential weighing, may have their merits in certain contexts, the use of sampling weights provided by the DHS program is considered appropriate and standard for analyzing data from multiple countries within the DHS framework. To address your concern, we will clarify in our revised manuscript that we have followed the recommended practice of using sampling weights provided by the DHS program for our multi-country analysis. Thank you for bringing this to our attention.**

7. DHS data may have different formats, standards, definitions, or classifications that make them difficult to compare, link, or aggregate across countries. How do authors handle the above challenges during multicounty analysis?

**Response: Dear Reviewer, thank you for your feedback on our study. We appreciate your comment regarding the potential challenges in comparing, linking, or aggregating DHS data across multiple countries. We understand your concern and would like to address it. We acknowledge that the DHS data may have variations in formats, standards, definitions, or classifications across countries. However, it is important to note that the DHS program has implemented measures to ensure consistency and comparability of the data across different countries.**

**The DHS program follows standardized protocols and guidelines for data collection, which include consistent survey instruments, questionnaires, and procedures. These protocols aim to minimize differences in data collection processes across countries, thereby facilitating comparability. In our study, we have employed rigorous procedures to handle the challenges associated with multi-country analysis. We have carefully reviewed and standardized the data formats, definitions, and classifications to ensure consistency and comparability across countries.**

**Additionally, we have appended the country-specific data files based on the enumeration area numbers (from the smallest to the largest EAs), following a logical and systematic approach. This approach enables us to organize the data in a consistent and coherent manner, facilitating accurate analysis and interpretation.**

**Furthermore, we have referenced the DHS program's guidelines and documentation to ensure the correct interpretation and application of variables and indicators, including the definition of exclusive breastfeeding.**

**In our revised manuscript, we will provide explicit details on the steps taken to address the challenges of comparing, linking, and aggregating DHS data across multiple countries. This will help readers understand our approach and the measures we have taken to ensure the accuracy and reliability of our analysis. Thank you for bringing this to our attention.**

8. It is good that the authors appended the West African countries DHS data for secondary analysis, but the big concern is how authors address the effect of the variation of time to combine data sets for analysis.

**Response: Dear reviewer, thank you for your feedback on our study. We appreciate your comment regarding the potential effect of combining data sets from West African countries that may have variations in the time of data collection. We understand your concern and would like to address it.**

**In our study, we acknowledge the potential impact of the variation in data collection time as a limitation in the discussion section. We recognize that there may be temporal differences in factors that could influence the outcomes under investigation. While it is true that the data sets from different countries may have been collected at different times, we have taken several factors into consideration when combining the data sets for analysis. These factors include the similarity in data collection methods, survey instruments, and the relatively close time proximity between the data sets.**

**Moreover, we have reviewed relevant literature and studies that have successfully utilized similar approaches in combining and analyzing DHS data from multiple countries. These studies have demonstrated the feasibility and validity of combining data sets collected at different times when the contextual factors are similar. In our revised manuscript, we put further emphasize these considerations and provide additional discussion on the potential implications and limitations of combining data sets from different time periods. We ensured that readers have a clear understanding of the steps we have taken and the limitations associated with the time variation in our data sets. Thank you for your valuable feedback.**

9. Cluster analysis have to conducted to identify patterns and trends in the data, such as the characteristics and behaviors of different segments of the population; explore the heterogeneity and diversity of the data; evaluate the impact and effectiveness of interventions or programs, such as the differences in outcomes or indicators across clusters; and inform policy and decision-making, such as the allocation of resources or the targeting of interventions to specific clusters.

**Response: Dear reviewer, thank you for your feedback regarding the cluster-based analyses and their recommendations to the policy makers and implementor. We have put all the recommendation based on our findings in the conclusions section. Thank you.**

10. Did the authors do sensitivity analysis? If not, I recommend the authors do a sensitivity analysis. Because it is essential to testing the robustness and reliability of the results of a data analysis by varying the assumptions, parameters, or data used in the analysis. Additionally Sensitivity analysis can help the authors to assess the impact of uncertainty, variability, or bias on the estimates or conclusions of their analysis.

**Response: Dear reviewer, thank you for your suggestion to perform a sensitivity analysis for the multilevel analysis. We appreciate your feedback and would like to elaborate on our rationale for not including a sensitivity, and or a receiver operating characteristic (ROC) analysis in our study. In our research, we focused on comparing the random effects and conducting model comparisons using several measures, including the proportional change in variance (PCV), intraclass correlation coefficient (ICC), median odds ratio (MOR), log-likelihood ratio (LLR), AIC, and deviance information criterion (DIC). These measures are commonly used in multilevel modeling to assess the goodness of fit and compare different models.**

**We believe that these model comparison metrics provide sufficient information to assess the fit of the models and compare them against each other. They allow us to evaluate the extent to which the inclusion of various determinants improves the model fit and captures the variability in the outcome variable. While ROC/sensitivity/ analysis is a valuable tool for evaluating the performance of prediction models and assessing sensitivity and specificity, we believe it is not as relevant in the context of our study. ROC analysis is typically used to evaluate the predictive accuracy of models and determine the optimal cutoff points for classification. However, in our study, our main objective was to identify the determinants of early initiation of breastfeeding rather than developing a predictive model. Moreover, in multilevel modeling, the focus is often on capturing the variation between clusters and assessing the impact of different level-2 predictors. Measures such as ICC, MOR, LLR, and DIC are well-established and widely used in this context to evaluate model fit and compare different models. We acknowledge that including a sensitivity analysis using ROC analysis could be valuable in some contexts, particularly when evaluating prediction models. However, in our specific research focus and given the aims of our study, we believe that the model comparison measures we used (PCV, ICC, MOR, LLR, and DIC) are sufficient to demonstrate the goodness of fit and compare the models.**

**We appreciate your suggestion and your consideration of including a sensitivity analysis. However, in light of the specific objectives and context of our study, we are confident that the model comparison measures we utilized provide a robust assessment of the models' fit and performance (refer the multilevel regression analysis tables please). Thank you for your valuable feedback.**

11. The results of the outcome variable and some independent variables are better represented in figures or charts than in tables.

**Response: Dear reviewer, we would like to inform you that we have addressed your request to present all independent variables in table form, as requested. Additionally, we have included the results of the outcome variable in chart form. Thank you very much for your valuable suggestion.**

Reviewer #2: General comment
The title is interesting and essential for the reduction of neonatal and children morbidity and mortality in the world. It is suitable to the journal of plose one.

**Response: Dear reviewer, thank you for your feedback on the title of our study. We appreciate your positive comments and agree with your assessment that the title is relevant and important for addressing neonatal and child morbidity and mortality worldwide. We are pleased to hear that you find the title suitable for the journal PLOS One. We believe that the chosen title effectively conveys the main focus and significance of our study. It captures the essence of our research and highlights the potential impact it can have on reducing neonatal and child morbidity and mortality rates globally. We are grateful for your positive evaluation of the title and your recognition of its relevance to the field. We will ensure that the manuscript aligns with the journal's guidelines and requirements. Thank you for your valuable feedback.**

Specific comments

1. Title: it is better to rearrange the title as “Determinants of early initiation of breastfeeding following to birth in West Africa: further analysis using multilevel model from multi country national health survey”. Why you said maternal and child related determinants???

**Response: Dear reviewer, thank you for your suggestion to rearrange the title of our study. We appreciate your input and understand your rationale for proposing the alternative title: "Determinants of early initiation of breastfeeding following birth in West Africa: further analysis using multilevel modeling from multi-country national health surveys." We agree that the revised title you proposed provides a clearer and more specific description of our study. It accurately highlights the key elements of our research, including the focus on determinants of early initiation of breastfeeding, the geographical context of West Africa, and the analytical approach utilizing multilevel modeling and data from national health surveys.**

**Regarding your question about the mention of "maternal and child related determinants," we apologize for any confusion caused. Upon reviewing our manuscript, we recognize that the term "maternal and child related determinants" may not accurately reflect the content of our study. We acknowledge that the determinants we explore are specifically related to early initiation of breastfeeding and may not encompass all aspects of maternal and child health. Therefore, we made the necessary revisions to ensure that the title accurately reflects the scope of our study, focusing on the determinants of early initiation of breastfeeding within the West African context.**

2. Introduction: on the introduction part of your study various determinant factors of early initiation of breast feeding were explored by various international reports and studies. What is the significance of this study particularly using DHS?

**Response: Dear reviewer, thank you for your comment regarding the significance of our study, particularly in relation to the use of Demographic and Health Survey (DHS) data, and the exploration of determinant factors of early initiation of breastfeeding. While it is true that various international reports and studies have examined the determinants of early initiation of breastfeeding, our study adds value by utilizing DHS data for West African countries. The DHS program collects comprehensive and nationally representative data on various health and demographic indicators, including breastfeeding practices, from a large number of countries.**

***The existing body of research on EIBF in West Africa is limited in several ways. Firstly, the number of studies conducted on this topic is scarce, indicating a lack of comprehensive understanding of the factors influencing EIBF in the region. Moreover, these studies have often focused on only a small portion of the population, limiting the generalizability of their findings to the broader West African context. Furthermore, previous studies have primarily examined individual-level factors associated with EIBF, such as maternal characteristics or infant health indicators. While these individual-level factors are undoubtedly important, there is a need to explore and understand the broader implementation factors that influence EIBF practices in West Africa. These implementation factors could include community-level dynamics, healthcare systems, cultural practices, and policy environments that may impact the initiation of breastfeeding within the region. To address these research gaps, the aim of our study was to assess the possible implementation factors influencing EIBF in West Africa. By taking a broader perspective and considering factors beyond the individual level, we sought to provide a more comprehensive understanding of the determinants and barriers to EIBF in the region. The scientific findings of this study will help the West African government and other stakeholders, policymakers and program implementers who have a lot of work to improve the reduced EIBF by developing a timely policy***

**By utilizing DHS data, our study benefits from its wide coverage and standardized methodology, allowing for cross-country comparisons and analysis of breastfeeding practices in West African countries. This enables us to explore the determinants of early initiation of breastfeeding within this specific regional context. Furthermore, our study contributes to the existing literature by specifically focusing on West African countries. We acknowledge that there is limited research conducted on this topic within the context of West Africa. Therefore, our study helps to fill this gap in knowledge and provides valuable insights into the factors influencing early initiation of breastfeeding in this region. The significance of our study in utilizing DHS data to explore the determinants of early initiation of breastfeeding, particularly within the context of West African countries where research has been limited. Thank you for raising this point, and we appreciate your valuable feedback.**

3. Methods: well-expressed and written

**Response: Dear reviewer, thank you for your validation**

4. Result : from table 2 under the variable educational status there is poor ,middle and rich

categories. What is this?

**Response: Dear reviewer, thank you for pointing out the confusion regarding the variable "educational status" in Table 2. We apologize for the error in labeling. To clarify, the categories mentioned as "poor," "middle," and "rich" under the variable "educational status" in Table 2 were mislabeled. This was an oversight on our part, and we sincerely apologize for any confusion caused. The correct variable under consideration should have been the "wealth index" instead of "educational status." The wealth index is a composite measure of household wealth based on various indicators such as household assets, housing conditions, and access to basic services. Typically, the wealth index is divided into categories such as "poorest," "poorer," "middle," "rich," and "richest" to represent different levels of household wealth.**

5. Discussion: well written

**Response: Thank you in advance for your feedback.**
