# Supplementary material for: Determinants of early initiation of breastfeeding following birth in West Africa: A multilevel analysis using data from multi-country national health surveys
Source: PLoS One. 2024 May 16;19(5):e0302143. doi: 10.1371/journal.pone.0302143 (PMC11098389; doi:10.1371/journal.pone.0302143)
Supplement: Attachment — Submitted filename: Reviewer comment.docx [file pone.0302143.s003.docx]

Reviewer comment:

General comment

The title is interesting and essential for the reduction of neonatal and children morbidity and mortality in the world. It is suitable to the journal of plose one.

**Specific comments**

1. **Title**: it is better to rearrange the title as “Determinants of early initiation of breastfeeding following to birth in West Africa: further analysis using multilevel model from multi country national health survey”. Why you said maternal and child related determinants???
2. **Introduction:** on the introduction part of your study various determinant factors of early initiation of breast feeding were explored by various international reports and studies. What is the significance of this study particularly using DHS?
3. **Methods:** well expressed and written
4. **Result :** from table 2 under the variable educational status there is poor ,middle and rich categories. What is this?
5. **Discussion:** well written
